# The effectiveness of Sustained Natural Apophyseal Glide on Flexion Rotation Test, pain intensity, and functionality in subjects with Cervicogenic Headache: A Systematic Review of Randomized Trials

**DOI:** 10.1186/s40945-022-00144-3

**Published:** 2022-09-01

**Authors:** Ricardo Cardoso, Adérito Seixas, Sandra Rodrigues, Isabel Moreira-Silva, Nuno Ventura, Joana Azevedo, Filippo Monsignori

**Affiliations:** 1 Escola Superior de Saúde, Universidade Fernando Pessoa, Porto, Portugal; 2 Transdisciplinary Center of Consciousness Studies of Fernando Pessoa University, Porto, Portugal; 3 Portugal LABIOMEP, Faculdade de Desporto, INEGI-LAETA, Universidade Do Porto, Porto, Portugal; 4 Faculdade de Desporto, CIAFEL, Universidade Do Porto, Porto, Portugal

**Keywords:** Physiotherapy, Cervicogenic headache, Sustained natural apophyseal glide, SNAG, Randomized controlled trials

## Abstract

**Objective:**

To determine the effect of sustained natural apophyseal glide (SNAG) on Flexion Rotation Test, pain intensity, and functionality in subjects with Cervicogenic Headache (CH).

**Methods:**

The research was conducted on five computerized databases PubMed/Medline, Web of Science, PEDro, Lilacs, and Cochrane Library (CENTRAL)*,* using the keywords combination: (sustained natural apophyseal glide OR SNAG OR joint mobilization OR Mulligan) AND (cervicogenic headache) according to PRISMA guidelines. The methodological quality of the included studies was analyzed using the Physiotherapy Evidence Database (PEDro) scale.

**Results:**

Eight articles fulfilled the eligibility criteria and were included in the review. The selected studies had a methodological quality of 6.6/10 on the PEDro scale and included a total of 357 participants. The SNAG significantly improved pain, Flexion Rotation Test and reduced functional symptoms.

**Conclusion:**

The available evidence suggests that SNAG may be a relevant intervention for CH.

**Supplementary Information:**

The online version contains supplementary material available at 10.1186/s40945-022-00144-3.


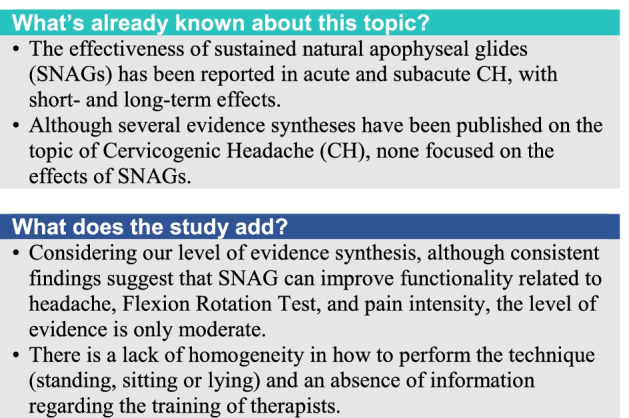


## Introduction

The International Classification of Headache Disorders defines cervicogenic headache (CH) as a “headache caused by a disorder of the cervical spine and its bone components, discs and/or soft tissues, usually but not invariably accompanied by neck pain” [1]. CH represents 15 to 20% of all headaches and is sometimes related to the abnormal activity of motor units in the scapular and neck muscles [2]. The dominant characteristics of CH generally include unilateral headache, pain induction with external pressure on the ipsilateral upper neck, limited cervical range of motion and triggering of symptoms by various clumsy neck movements [3, 4]. Weakness in the deep neck flexors and stiffness of the upper trapezius, levator scapulae and sternocleidomastoid muscles are common clinical findings in patients with CH [5].

CH intervention includes preventive approaches, anesthetics, denervation procedures and surgery. The choice of treatment must be made based on the individual's characteristics [6]. Physiotherapy is commonly used to treat CH [7], namely with spinal manipulative therapy, including mobilization and manipulation [8]. The concept of “mobilization with movement”, known as Mulligan's Concept, has distinct characteristics when compared with other forms of manual therapy, combining manual techniques with active movement. Sustained Natural Apophyseal Glides (SNAG) are one of the techniques used by the Mulligan concept. Mulligan described this technique as a painless glide applied in the joint, while the patient performed an active movement in the direction of the symptoms [9]. The effectiveness of SNAG in C1-C2 has been reported in patients with acute and subacute CH, with short- and long-term effects due to gate control theory and by correcting the "positional fault". “Positional fault” is a condition in which a joint is not resting in a neutral position and causes restrictions in physiological movement [10, 11].

Although previous systematic reviews [10, 11] presented numerous treatment techniques, they did not focus on analyzing the effects of SNAG on CH. In addition, other studies have been published on this topic.

To our knowledge, to date, there is no published evidence synthesis based on clinical trials for the effects of SNAG on CH. Therefore, the aim of this systematic review is to summarize and synthesize the clinical evidence on the effects of SNAG on Flexion Rotation Test, pain intensity, and functionality in subjects with CH.

## Main text

### Methods

We conducted this systematic review in accordance with the Preferred Reporting Items for Systematic Reviews and Meta-Analyzes (PRISMA), which aims to improve the presentation standards for systematic reviews and meta-analyzes [12].

#### Construction of the research question

PICO represents an acronym for Patient, Intervention, Comparison and Outcome. Table 1 presents the four components of PICO strategy and the construct of the research question.

**Table 1:** Description of the components in PICO strategy for the systematic review

Acronym	Definition	Description
P	Patient	individuals with Cervicogenic Headache
I	Intervention	Sustained Natural Apophyseal Glide
C	Comparison	control, placebo or other standard intervention
O	Outcome	Flexion Rotation Test, pain intensity and functionality

We conducted the computerized search in PubMed/Medline, Web of Science, PEDro, Lilacs and Cochrane Library (CENTRAL) databases to find studies that assessed the effects of the Mulligan SNAG technique on subjects with cervicogenic headache, published until May 2022. The following combination of keywords was used: (sustained natural apophyseal glide OR SNAG OR joint mobilization OR Mulligan) AND (cervicogenic headache) on PubMed/Medline, Web of Science, Lilacs and Cochrane Library (CENTRAL) databases. The search strategy was adapted to meet the requirements of PEDro database and a combination of each of the search terms related to the technique (sustained natural apophyseal glide / SNAG/ joint mobilization / Mulligan) and the search term related to the condition (cervicogenic headache) was used. The lists of references from the included studies were also screened for any additional study.

Our inclusion criteria were: (1) Randomized controlled trials (RCTs); (2) randomized clinical trials; (3) studies performed in humans; (4) published until May 2022; (5) written in English, Italian, French, Spanish or Portuguese; (6) with reference to SNAG technique in cervicogenic headache; (7) assessed Flexion Rotation Test, or/and pain intensity measured by any scale or test, or/and functionality (using the following scales: Headache Disability Inventory; the Dizziness Handicap Inventory; the Neck Disability Index; the Marginal Means-Headache Index). The exclusion criteria were: (1) books; (2) used SNAG in a different headache type (3) score less than 5 on the PEDro scale.

Two independent reviewers implemented the search strategy and screened the titles and abstracts to identify studies that potentially meet the eligibility criteria. The full text of the potentially eligible studies was retrieved and assessed independently by the same reviewers for compliance with the defined eligibility criteria. In case of disagreement over the eligibility of specific studies, a third reviewer was involved, and consensus was obtained.

#### Data extraction

Two reviewers conducted the data extraction. The characteristics of the collected studies included the authors, year of publication, sample size, study design, outcome measures, and results. The outcome measures selected were Flexion Rotation Test, pain intensity and Functionality.

##### Flexion Rotation Test

This passive examination procedure involves fully flexing the cervical spine so that the vertebral movement is theoretically constrained to C1- C2, then assessing the cervical rotation range of motion in this position. The normal range of motion is 44° to each side [13].

##### Pain intensity

Usually assessed through the Visual Analogue Scale (VAS) which is a measurement instrument that tries to measure a characteristic or attitude that is believed to range across a continuum of values and cannot easily be directly measured.

##### Functionality

Headache is associated with varying levels of symptoms that cannot be assessed adequately in the context of the conventional clinical evaluation; for this reason, many studies use functionality questionnaires (Headache Disability Inventory; the Dizziness Handicap Inventory, the Neck Disability Index; the Marginal Means-Headache Index) as an evaluation parameter to measure disabilities due to CH.

#### Data analysis

Heterogeneity was assessed from a methodological (study methodology) and clinical (sample characteristics) perspective and, considering the differences between the included studies, the results were not pooled using a metanalytic approach. Instead, a level of evidence synthesis was conducted for each assessed outcome (Flexion Rotation Test, pain, and functionality). If at least 75% of the studies analyzing an outcome point in the same direction, i.e. 75% demonstrate improvements or worsening of the symptoms, the findings were considered consistent. The level of evidence was defined as “strong” if consistent findings were found in high-quality studies. The level of evidence was considered moderate if the results, in at least 66% of the studies point in the same direction and were high quality studies, or if in at least 75% of the studies point in the same direction and were moderate quality studies. If an outcome was investigated only in a single high-quality study the level of evidence was considered “limited”. If the results reveal inconsistent findings, the level of evidence was considered inconclusive [14].

#### Methodological quality and risk of bias

The methodological quality of each RCT included in this review was assessed by two independent reviewers using the Physiotherapy Evidence Database (PEDro) scoring scale [15]. The PEDro scale consists of a checklist of 11 criteria, of which only 10 criteria are scored. For each criterion that the study met, 1 point was awarded. Clear and unambiguous fulfillment of a criterion leads to the award of 1 point. Thus, a total of 10 points are available. The scale applies only to experimental studies. The PEDro scale does not assess clinical utility [15]. Disagreements in scoring were resolved by the reviewers in an oral discussion. A consensus was reached on all studies at the first meeting. Studies were considered to be of moderate quality if the score ranged from 5–7 and of high quality if the score ranged from 8–10 because according to the PEDro database website, a score of 8 is optimal for complex interventions.

## Results

### Studies selection

**Fig. 1:**
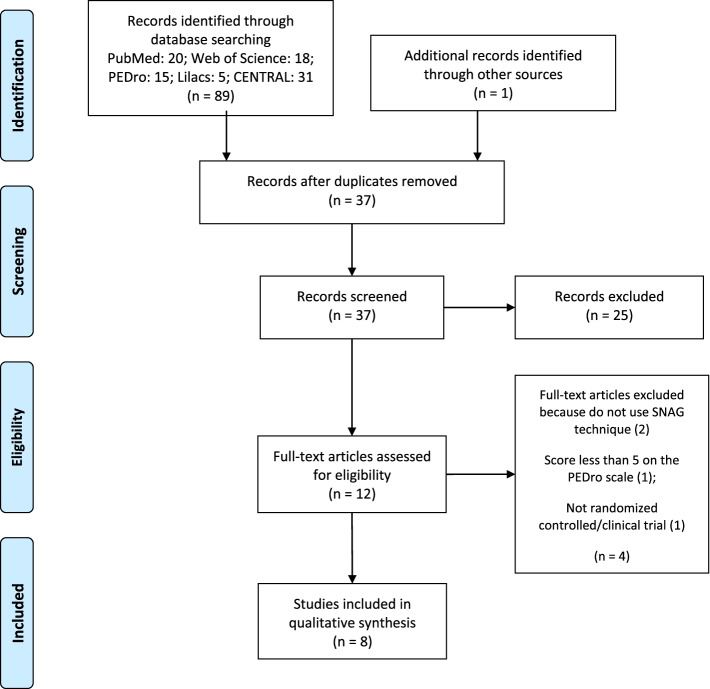
PRISMA flow diagram of the literature selection process

Our electronic search identified 89 records and we added one additional record [16] after reading references of the included studies [17]. After the removal of duplicates, two independent reviewers screened 37 studies and excluded 25 reports mainly because do not respondent to inclusion criteria by reading titles and abstracts. The same two reviewers retrieved and read in detail the full text of 12 articles. After careful analysis, 4 studies were excluded because did not use SNAG technique. Finally, we included 8 studies in the systematic review (Fig. 1).

### Description of the included studies

The included studies represented a total sample size of 357 participants (minimum sample size of 23 and a maximum of 114), aged between 25 and 60 years. The included articles were summarized in the following table of contents (Table 2).

**Table 2:** Summary of included studies

Authors (years)	Objective of the study	Sample size / therapist training / study design	Treatment Method / treatment period and evaluation	Outcome measures	Results
**Hall et al. ( 2007) **[18]	To investigate the efficacy of the treatment of C1-C2 self-SNAG and the effects on the change of FRT and symptoms related to cervicogenic headache	*n* = 32 13 M; 19 F MA: 36 years / Performed by 3 professionals/ RCT-Parallel	Trial Period: 12 months CG: False mobilization of C1-C2 using the cervical self-SNAG strap 2 times a day SG1: Self mobilization (self-SNAG) of C1-C2 using the cervical self-SNAG strap 2 times a day	FRT MMHI	CG: ↑ FRT ( *p* < 0.001); = MMHI SG1: ↑ FRT ( *p* < 0.001); ↑ MMHI( p < 0.001) FRT: SG1 > CG MMHI: SG1 > CG
**Shin and** **Lee, (2014)** [19]	To investigate the effect of the SNAG technique on the duration of headache in middle-aged women with CH	*n* = 40 37 F MA: 45 years / Performed by 3 professionals / RCT-Parallel	Trial Period: 7 weeks CG: Placebo mobilization of the neck area with pain—3 × week (20 min) for 4 weeks SG1: SNAG technique—3 × week (20 min) for 4 weeks	VAS NDI HD	CG: ↑ VAS ( *p* < 0.05); ↑ HD ( *p* < 0.05); ↑ NDI ( p < 0.05) SG1: ↑ VAS ( *p* < 0.05); ↑ HD ( p < 0.05); ↑ NDI ( *p* < 0.05) VAS: SG1 > CG NDI: CG = SG1 HD: SG1 > CG
**Wade and Franklin, (2015) **[16]	To study the effects of the SNAG technique and strengthening muscles in the cervical spine in the relief of cervicogenic headache	*n* = 30/ M;F (Not specified) MA: 35 years/ Performed by 2 professionals/ RCT-Parallel	Trial Period: 1 week CG: TENS in the paraspinal region of the cervical spine combined with deep muscle strengthening for the cervical region SG1: C1-C2 SNAG 10 × 10 s, combined with deep muscle strengthening for the cervical region	VAS SDCM	GC: ↑ VAS( *p* < 0.000); SDCM ( *p* < 0.000) SG1: ↑ VAS( *p* < 0.000); SDCM( p < 0.000) VAS: SG1 > CG SDCM: SG1 = CG
**Christian (2017)** [20]	Search for the effects of SNAG (Mulligan) and PA (Maitland) techniques on cervicogenic headache and compare them with a control group	*n* = 23 12 M; 11 F MA: 30 years/ (Not specified)/ RCT-Parallel	Trial Period: 1 week CG: n = 6 sessions (active neck exercises + strengthening and stretching exercises) SG1: n = 6 SNAG sessions (4 × 10 s.) SG2: n = 6 sliding sessions central PA, lateral PA (from 3 × 60 to 5 × 120 oscillations)	FRT HDI	CG: ↑ FRT ( *p* < 0.05) SG1: ↑ FRT ( *p* < 0.05); SG2: ↑ FRT ( *p* < 0.05) FRT: CG = SG1 = SG2 HDI1: CG > SG2
**Patra, Mohanty,** **Gautam (2017)** [17]	Evaluate the effectiveness of dry needling combined with SNAG C1-C2 in improving PPT and reducing disability due to cervicogenic headache	*n* = 114 37 M; 77F MA: 37 years/ Performed by 3 professionals/ RCT-Parallel	Trial Period: 6 weeks SG1: Dry Needling SG2: C1-C2 SNAG SG3: GE1 + GE2	HDI PPT-1 PPT-2 PPT-3	SG1: ↑ HDI ( *p* < 0.001); ↑ PPT-1 ( *p* < 0.001); ↑ PPT-2 ( *p* < 0.001); ↑ PPT-3 ( *p* < 0.001); SG2: ↑ HDI ( *p *< 0.001); ↑ PPT-1 ( *p* < 0.001); SG3: ↑ HDI ( *p* < 0.001); ↑ PPT-1 ( *p* < 0.001); ↑ PPT-2 ( *p* < 0.001); ↑ PPT-3 ( *p* < 0.001); HDI: SG3 > SG2 > SG1 PPT-1: SG3 > SG2 > SG1 PPT-2: SG3 > SG1 > SG2 PPT-3: SG3 > SG1 > SG2
**Kirthika et al. (2018)** [21]	To compare the effects of the SNAG and MET techniques on the life management of subjects with cervicogenic headache	*n* = 30 6 M; 24 F MA: 26 years/ Performed by 4 professionals/ RCT-Parallel	Trial Period: 4 weeks SG1: SNAG 3 sets of 5 repetitions SG2:MET 6 sets de 5 repetitions	VAS HDI	SG1: ↑ VAS ( *p* < 0.001); ↑ HDI2 ( *p* < 0.001) SG2: ↑ VAS ( *p* < 0.0001); ↑ HDI2 ( *p* < 0.001) VAS: SG2 > SG1 HDI2: SG2 > SG1
**Mohamed et al. (2019)** [22]	To determine the effect of the SNAG technique on C1-C2 on cervicogenic headache and associated dizziness	*n* = 48 28 M; 20F MA: 29 years/ Performed by a therapist/ RCT-Parallel	Trial Period: 6 months SG1: SNAG C2 (10 × 10 s. / 30 s rest each repetition) SG2: C1-C2 SNAG Half-Rotation (10 × 10 s / 30 s of rest each repetition) SG3: Combination of GE1 + GE2 (5 repetitions of each technique)	NDI DHI FRT	SG1: ↑ FRT (p < 0.001); ↑ DHI (*p* < 0.001) SG2: ↑ FRT (*p* < 0.001); ↑ DHI (*p* < 0.001) SG3: ↑ NDI (*p* < 0.001); ↑ FRT (*p* < 0.001); ↑ DHI (*p* < 0.001) NDI: SG3 > SG1 = SG2 FRT: SG3 > SG1 = SG2 DHI: SG1 > SG3 > SG2
**Kashif et al. (2021) **[23]	To investigate the effect of SNAGs in the treatment of cervicogenic headache	*n* = 40 0 M; 40 F MA: 22 years/ Performed by a manual therapist/ RCT-Parallel	Trial Period: 4 weeks SG1: 10 × 10 s; 3 × week (20 min; 12 sessions) CG: received placebo treatment (contact pressure of the hand touching the disturbed joint) 10 × 10 s; 3 × week (20 min; 12 sessions)	VAS (assessed on week 1, 2, 3 and 4)	SG1: ↑ VAS ( *p* < 0.001) VAS: SG1 > CG on week 3 ( *p* < 0.001) and week 4 ( *p* < 0.001)

*Code*:↑—improvement; >—significantly better; *DHI* Dizziness Handicap Inventory, *F* Female, *FRT* Flexion-Rotation Test, *HD* Headache Duration, *HDI* Headache Disability Inventory, *HDI* Headache Disability Index, *M* Male, *MET* Muscular Energy Technique, *MA* Mean age, *MMHI* Marginal Means-Headache Index, *NDI* Neck Disability Index, *PPT* Pressure Point Threshold, *PPT1* Pressure Point Threshold Over Suboccipital Area, *PPT2* Pressure Point Threshold Over C5-C6 Paraspinal Area, PPT3 Pressure Point Threshold Over the Trapezius Muscle, *SDCM* Strength Deep Core Muscle, *TENS* Transcutaneous Electrical Nerve Stimulation, *VAS* Visual Analogic Scale

Three out of eight studies [18, 20, 22] analyzed the dysfunction in the C1-C2 segment performing the Flexion Rotation Test (FRT) as a reference test. Five trials assessed pain, four assessed pain intensity through the Visual Analogue Scale (VAS) [16, 19, 21, 23] and one through pressure pain threshold. [17] The functionality was assessed with the Headache Disability Index, also called Headache Disability Inventory (HDI) [17, 20, 21], the Dizziness Handicap Inventory (DHI) [22], the Neck Disability Index (NDI) [19, 22] and the Marginal Means-Headache Index (MMHI) [18] to assess self-perception of the disabling effects caused by headaches and their treatment in daily life.

Regarding the results, in the study by Christian (2017) [20], after a week of treatment, the group of SNAG showed a significant redution of Headache Disability Index when compared to the control group. The study by Shin and Lee (2014) [19] showed that SNAG significantly reduced pain and headache duration, when compared to a sham manual treatment. The study by Kirthika et al. (2018) [21] lasted for four weeks, and SNAG group, immediately post treatment, improved Headache Disability Index and reduced pain. However, muscle energy techniques (MET) group presented significant improvements in theese outcome measures, when compared to SNAG group. In the study by Patra, Mohanty and Gautam (2017) [17] the group that comprised the treatment with a combination of SNAG and dry needling, achieved significant improvements after six weeks, compared to the other two groups. The study by Wade and Franklin (2015) [16] revealed that the group of SNAG and strengthening of the cervical muscles through biofeedback showed significant improvements regarding the control group. Considering long term results, the study by Mohamed et al. (2019) [22], which lasted six months, there were significant differences between the experimental groups, where the group that received a combination of SNAG + Half-Rotation SNAG techniques demonstrated better results. Finally, in the study by Hall et al. (2007) [18], the experimental group that applied therapeutic self-mobilization techniques using the self-SNAG belt, showed significantly higher differences compared to the control group that applied “false” auto-SNAG techniques.

### Methodological quality and risk of bias of included studies

**Fig. 2:**
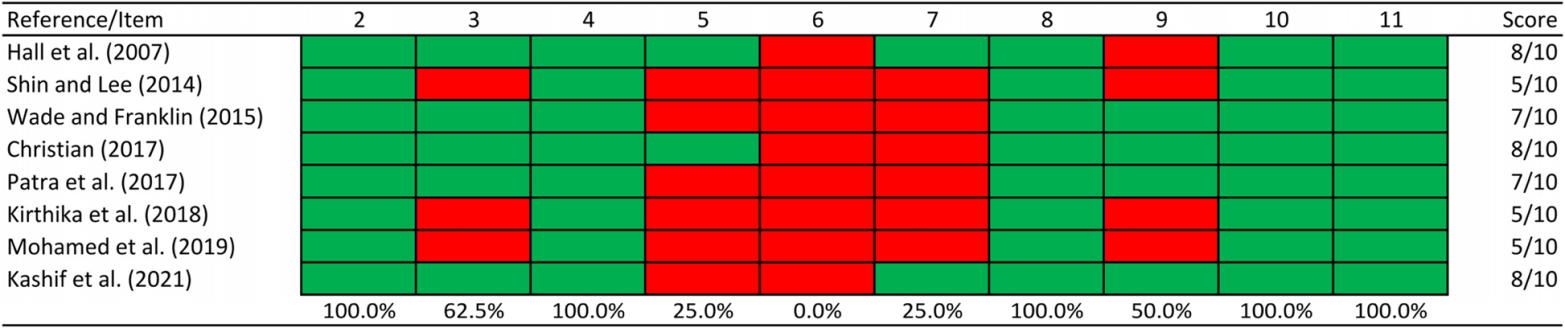
Methodological quality assessment of the included studies with PEDro scale. (2) Subjects were randomly allocated to groups (in a crossover study, subjects were randomly allocated an order in which treatments were received); (3) allocation was concealed; (4) the groups were similar at baseline regarding the most important prognostic indicators; (5) there was blinding of all subjects; (6) there was blinding of all therapists who administered the therapy; (7) there was blinding of all assessors who measured at least one key outcome; (8) measures of at least one key outcome were obtained from more than 85%; of the subjects initially allocated to groups; (9) all subjects for whom outcome measures were available received the treatment or control condition as allocated or, where this was not the case, data for at least one key outcome was analysed by “intention to treat”; (10) the results of between-group statistical comparisons are reported for at least one key outcome; (11) the study provides both point measures and measures of variability for at least one key outcome

The mean PEDro score of the included studies in the review was 6.6 (± 1.4; range: 5–8) points. Three studies were considered to have high methodological quality and five studies were considered to have moderate methodological quality (Fig. 2).

There was not a high degree of variation in quality between studies, however only two studies was able to satisfy the blinding criteria for the assessors (PEDro scale question 7), no studies were able to satisfy the blinding criteria for the therapists (PEDro scale question 6), six RCTs were not able to satisfy the blinding criteria for the subjects (PEDro scale question 5), four studies were not able to satisfy the PEDro scale question 9 and three investigations were not able to satisfy the allocation criteria (PEDro scale question 3). The remaining criteria were always scored positively. Given the difficulty to comply with the blinding criteria given the nature of the intervention performed, it is unsurprising that there were difficulties to apply it. Therefore, performance bias was the greatest risk of bias of the included studies, given the inappropriate blinding of patients (75%), outcome assessors (75%) and therapists (100%). Concerns with selection bias were detected in three studies, which failed to report proper allocation of participants (37.5%). Some concerns related to the lack of reporting of intention to treat analysis (attrition bias) exist in four studies, however, since all studies obtained outcome measures from more than 85% of the subjects initially allocated to groups and nothing was explicitly reported regarding deviations from intended interventions, we do not consider this fact as high risk of bias of the study results.

## Discussion

The presence of CH is a clinical condition that is often found in physiotherapy settings, so it is necessary to take into account the different treatment strategies and techniques used during clinical practice to promote the deepening of knowledge related to this area. In this review, the effects of Mulligan's SNAG technique on symptoms related to CH were evaluated. As described in Table 2, some studies have shown the effectiveness of the SNAG technique in improving important parameters such as increasing range of motion, assessed in the studies of Christian (2007) [20] and Kirthika et al. (2018) [21] and decreasing in functional symptoms that interfere with daily life, as described in the studies by Hall et al. (2007) [18], Mohamed et al. (2019) [22], Kirthika et al. (2018) [21], Shin and Lee (2014) [19], Kashif et al. (2021) [23], and Patra, Mohanty and Gautam (2017) [17]. However, considering our level of evidence synthesis, although consistent findings suggest that SNAG can improve functionality related to headache, FRT, and pain intensity, the level of evidence is moderate.

Therefore, the application of SNAG seems to positively influence important outcomes in these subjects. This positive effect may be related to a decrease in the excessive reactivity of the cervical nuclei of the trigeminal nerve and blocking the stimuli of the A-beta fibers, which can result in pain and disability relief [24].

Another possible explanation may have to do with the “Gate Control” theory, which states that the stimulation of mechanoreceptors in the joint capsule and the surrounding tissues may cause inhibition of pain in the spinal cord [25, 26]. In addition, descending pain-inhibiting pathways can also be activated, mediated by areas such as the periaqueductal gray matter of the midbrain. The final positioning in the rotation with SNAG may involve these inhibition and pain reduction systems [27].

According to the studies by Wade and Franklin, (2015) [16] and Mohamed et al. (2019) [22], the treatment protocol using SNAG that appears to have better effects consists of placing the patient in a sitting position and performing 10 repetitions (glide) for 10 s, with a 30-s pause per repetition. This protocol proved to have beneficial effects in the short term (as the first study lasted for a week) and in the long term (as the second study lasted six months).

### Flexion rotation test

To assist in the diagnosis of cervicogenic headache and, in particular, C1-C2 segmental dysfunction, many studies have suggested using this manual test [28]. This last study also found that subjects with cervicogenic headache have an average of 17° less rotation toward the headache side in the FRT, in contrast to those with no headache or migraine with aura. The studies by Dvorak (1992) [13] and Ogince, Hall, Robinson and Blackmore (2006) [29] supported these findings. In the investigation carried out by Hall et al. (2007) [18] the self-SNAG technique showed significant differences in FRT compared to the control group. The amount of rotation improved in both the C1-C2 self-SNAG and placebo groups. However, this improvement was significantly greater following active treatment. Rotation increased by 15° to 39° in the C1-C2 self-SNAG group. In the study conducted by Christian (2017) [20], the SNAG technique improved the FRT as in the group with Maitland techniques and the active cervical exercise control group. However, the group that performed SNAG was the only one that significantly increased the range of motion. In another study, Mohamed et al. (2019) [22] compared several typologies of the technique, such as SNAG, SNAG Half-Rotation and the combination of the two. All groups had improvements in the FRT. However, at the end of the 6-month study period, the experimental group that had the SNAG technique as a treatment showed significant improvements compared to the other groups.

Two high quality studies [18, 20] reported significantly better results in the SNAG group and one moderate quality study [22] reported similar results in the SNAG and control groups. However, in this study, a significant improvement in FRT was observed in the SNAG group and the control group consisted in C1-C2 SNAG Half-Rotation.

### Pain intensity

The VAS was used in this study for the assessment of patients’ neck and shoulder joint pain. The reliability of this assessment tool is confirmed in the study reported by Bijur et al. (2001) [30]. In the investigation by Wade and Franklin (2015) [16], it was concluded that the SNAG group showed significant differences compared to the control group in VAS, indicating a decrease in the intensity and frequency of symptoms. The studies by Shin and Lee (2014) [19] and Kirthika et al. (2018) [21] used the VAS scale as an evaluation method; the first study showed that the SNAG technique significantly improved headache pain and duration compared to the placebo group. Finally, in the study by Kirthika et al. (2018) [21] all groups improved concerning the conditions measured at the beginning of the study. The group that applied MET revealed statistically significant improvement of VAS comparing to the group that performed SNAG.

One high quality and Two moderate quality studies [16, 19, 23] reported significantly better results in the SNAG group and two moderate quality studies [17, 21] reported significantly better results in the control group. Despite better results were achieved in the control group, Kirthika et al. [21] reported a significant improvement in pain in the SNAG group.

### Functionality questionnaires

All studies included in this review used functionality questionnaires as an evaluation parameter to measure the incapacitating effects and disabilities due to CH. The studies of Christian (2017) [20], Kirthika et al. (2018) [21] and Patra, Mohanty e Gautam (2017) [17] used the HDI (Headache Disability Index, also called Headache Disability Inventory) to assess headache related disability. The study conducted by Christian (2017) [20] showed that there is a decrease in the severity of a headache among all study groups, but it was greater in the manual therapy group (SNAG) than in control group. The second study showed a reduction of the mean value of DHI compared at the baseline at the end of the last treatment. Also, the NDI (Neck Disability Index) is used like a questionnaire designed to inform how neck pain has affected the ability to cope in daily life. Evaluation items included pain intensity, headache, concentration, pain intensity, lifting things, personal care and leisure activity [31]. The studies conducted by Shin and Lee (2014) [19] and by Mohamed et al. (2019) [22] used a NDI and showed improvements in the group performing SNAG; especially in the second study, the score decreased significantly after treatment in the three groups and post-treatment NDI was significantly lower in the group that used a combination of SNAG compared to the other two groups.

Two high quality studies [18, 20] and three moderate quality studies [17, 19, 22] reported significantly better results in the SNAG group, and one moderate quality study [21] reported significantly better results in the control group. In this study [21], significant improvements were observed in the SNAG group regarding this outcome.

A limited number of studies provide evidence regarding the effectiveness of SNAG on CH, with few RCT’s addressing the subject. Additionally, there is a lack of homogeneity in how to perform the technique (standing, sitting or lying) and an absence of information regarding the training of therapists.

### Limitations

This review has some limitations that should be considered. Although the databases included in the review are well established in the research community, the selection of search terms and databases, the absence of a grey literature review and review of reference lists, as well as the language restrictions may have limited the amount of studies included in the review. Another limitation is that we do not have a registered review protocol, which may have contributed to unplanned duplication and did not allow a comparison of reported and planned review methods.

Despite these limitations, this review is, as far as the authors are aware, the first to comprehensively and critically assess the evidence for the effects of SNAG on CH, which may provide useful insights for further studies.

To achieve the highest quality evidence, future studies should use high-quality RCT designs with large samples and adequate follow-up, and patients, therapists, and investigators should be properly blinded. These are the most common methodological problems identified in the included studies**.**

## Conclusion

The findings from the present review seem to suggest that the use of SNAG can reduce the symptoms associated with CH in the short, medium, and long term. However, it is important to emphasize that more studies are needed in this area of research. Regarding our level of evidence synthesis, although consistent findings suggest that SNAG may improve functionality related to headache, FRT and pain intensity, the evidence is moderate. Summing up, SNAG may be a relevant intervention to treat participants with cervicogenic headache**.**

## Supplementary Information

**Additional file 1. **PRISMA 2020 Checklist.

## Data Availability

The datasets used and/or analysed during the current study are available from the corresponding author on reasonable request.

## Abbreviations

CH: Cervicogenic Headache
DHI: The Dizziness Handicap Inventory
FRT: Flexion Rotation Test
HDI: Headache Disability Inventory
PEDro: Physiotherapy Evidence Database
PRISMA: Preferred Reporting Items for Systematic Reviews and Meta-Analyzes
MMHI: Marginal Means-Headache Index
MET: Muscle energy techniques
NDI: The Neck Disability Index
RCT: Randomized controlled trial
SNAG: Sustained natural apophyseal glide
VAS: Visual Analogue Scale
